# Comparison of Ultrasound and Computed Tomography Scanning Accuracy in Diagnosing Acute Appendicitis at King Abdulaziz University Hospital

**DOI:** 10.7759/cureus.31880

**Published:** 2022-11-25

**Authors:** Anas Raffa, Ahmed Abduljabbar, Ahmed Alharthy

**Affiliations:** 1 Department of Radiology, King Abdulaziz University Faculty of Medicine, Jeddah, SAU

**Keywords:** king abdulaziz university hospital (kauh), specificity, sensitivity, computed tomography, ultrasound, acute appendicitis, appendix

## Abstract

Background: Choosing the most effective and accurate preoperative modality is one of the most significant tools in the clinical diagnosis of acute appendicitis (AA) to prevent negative appendectomies, diagnosis confusion, and delayed treatment. This decision making remains challenging to emergency physicians and surgeons which lead this study to determine the sensitivity and specificity of ultrasound (US) and computed tomography (CT) in predicting AA using pathology reports as a reference at King Abdulaziz University Hospital (KAUH), Jeddah, Saudi Arabia.

Methods: This study was conducted retrospectively at the Emergency Department, KAUH in Jeddah, Saudi, Arabia using 351 medical records with a clinical picture of acute appendicitis and no history of trauma. The sensitivity and specificity were calculated for ultrasound and computed tomography imaging. The positive predictive values (PPV) and negative predictive value (NPV) were also evaluated.

Results: Out of the total 351 patients included in this study, 83 patients underwent surgical appendectomies and the pathology results revealed that 64 patients were diagnosed with AA while 19 showed a normal appendix. Of the 64 patients, 18 underwent US imaging while 62 underwent CT imaging. Compared to pathology results, US imaging results revealed that 12 out of 14 positive patients (85.7%) and only two out of four negative patients (50.0%) were correctly predicted while two out of 14 positive patients (14.3%) and two out of four negative patients (50.0%) were falsely diagnosed. On the other hand, CT imaging results revealed that 46 out of 49 positive patients (93.9%) and 9 out of 13 negative patients (69.2%) were correctly predicted while only three out of 49 positive patients (6.1%) and only four out of 13 negative patients (30.8%) were incorrectly diagnosed.

Conclusion: Having an accuracy of 88.71%, sensitivity of 92.00%, and specificity of 75.00%, CT imaging was found to be more effective and accurate than US imaging which was only 77.78% accurate, 85.71% sensitive, and 50.00% specific. Statistical analyses also revealed that US results have a significant difference with pathology results (P= 0.130) while CT result has no significant difference (P <0.001).

## Introduction

Appendicitis refers to the inflammation in the vermiform of the appendix [1]. Globally, it was estimated that there are 11 cases of acute appendicitis (AA) in every 10,000 population per annum [2]. In almost every emergency department, AA has been considered as the most prevalent cause of pain in the lower abdominal part of a suspected patient. In a general population, the overall risk and prevalence for AA is approximately 7% wherein it was estimated that the risk for males and females are 9% and 6%, respectively. Considering the available data for geographical differences, it was estimated that the lifetime risks for the USA, Europe, and Africa are 9%, 8%, and 2%, respectively [2-5]. Moreover, AA is believed to occur at any age but rarely occurs at extreme ages. Approximately, AA peak prevalence occurs between the age of 10 to 14 years for female patients while the age of 15 to 19 years for male patients [2,3].

For the past decades, appendectomy has been considered one of the most extensively performed types of treatment for appendicitis. Although it is generally tolerated, such surgical operation is still associated with postoperative morbidity and mortality [2]. Thus, it is very fundamental to achieve high accuracy in clinical diagnosis in order to provide better decision making as to whether appendectomy is necessary for a suspected patient or not [4]. The clinical diagnosis of AA can be done through the patient’s historical record, physical examinations, and laboratory tests. For patients with typical signs and symptoms, the diagnosis might be straightforward, but the presentation is often atypical which might lead to diagnosis confusion and treatment delay [3,6]. For some instances where the clinical diagnosis is questionable, preoperative imaging, namely US, CT, and magnetic resonance imaging (MRI), should be used [7]. For the past years, the US has been used as an important tool in diagnosing AA among suspected patients and as a basis for a clinical examination continuation [7]. Meanwhile, CT has been significant in decreasing the percentage of unnecessary appendectomies and was accepted as the standard for the evaluation of patients with suspected AA. Lastly, MRI has shown a high accuracy rate in AA detection and usefulness during the inapplicability of radiation, such as for pregnant women and young patients [8].

In this study, the effectiveness of CT and US in predicting AA at KAUH was evaluated. Specifically, it aimed to compare the sensitivity and specificity of the two radiological tests in predicting AA and compared their reliability against the pathology results of accurately diagnosed AA patients.

## Materials and methods

Study subjects and design

This study was conducted retrospectively at the Emergency Department, KAUH in Jeddah, Saudi, Arabia using 351 medical records with a clinical picture of acute appendicitis with no history of trauma. The most commonly observed clinical presentations included acute right iliac fossa pain, vomiting, nausea, positive sonographic murphys' sign and/or rebound tenderness. None of the collected data from this study can be associated with the patients’ personal identities. No personal identifier was used during the data collection and analysis. All patients were admitted between January 2016 and January 2019 and diagnosed with AA, regardless of their demographic data, such as age and gender. The total population comprised 127 males (36.2%) and 224 females (63.8%), with a mean age of 32.09 ± 17.3, minimum and maximum age of 4 and 83 years old, respectively. Two independent authors collected the data from the hospital's electronic medical records and radiology Picture Archiving and Communication Systems (PACS), evaluated for inclusion/exclusion criteria, and performed the analyses. Inclusion criteria were comprised of the suspected clinical picture of acute appendicitis who underwent either ultrasound imaging, computed imaging, or both, without an alternative diagnosis at discharge. Patients with alternative diagnoses based on imaging or histopathology, perforation on imaging, or treated as perforated acute appendicitis were excluded from the study. Histopathological results on tissue obtained from appendectomy were used as the standard for the final diagnosis of AA and for the evaluation of the effectiveness of US and CT scanning in determining AA. The sensitivity and specificity were calculated for both imaging techniques. The positive predictive values (PPV) and negative predictive value (NPV) were also evaluated. Meta-analyses were performed for relevant subgroups, and sensitivity analysis was completed to account for outliers.

Statistical methodology

This study was analyzed using SPSS version 23 (IBM Corp., Armonk, NY). A simple descriptive statistic was used to define the characteristics of the study variables through the form of counts and percentages for the categorical and nominal variables while continuous variables are presented by mean and standard deviations. All relevant parameters such as the accuracy, sensitivity, specificity, PPV, NPV, and disease prevalence are expressed as percentages. The confidence intervals (CI) for accuracy, sensitivity, and specificity were reported as "exact" Clopper-Pearson CI.

## Results

Female predominance at 63.8% was observed in the total population of included patients’ medical records (n= 351), giving only 36.2% for male distribution. The total population was classified according to four age groups. The mean age was 32.09 ± 17.3 years old with a minimum age of 4 and maximum age of 83. The data are shown in Table 1.

**Table 1:** Demographic data of patients (N= 351). SD: Standard Deviation, N: Number

Demographics	Type	Count	Percentage, %
Gender	Male	127	36.2
	Female	224	63.8
Age	≤20	102	29.1
	21-40	157	44.7
	41-60	64	18.2
	>60	28	8.0
	Minimum	4	
	Maximum	83	
	Mean ± SD	32.09 ± 17.03	

As presented in Table 2, 228 patients (65.0%) of the total population underwent US imaging. The US imaging results revealed that 22 patients were positive (6.3%), 54 were negative (15.4%), and 152 patients had inconclusive data (43.3%). With the same population, 226 patients (64.4%) underwent CT imaging. The CT results revealed that 79 patients were positive (22.5%), 141 were negative (40.2%), and six patients had inconclusive data (1.7%). The imaging showed that of the 64 patients with post-appendectomy pathology-proven acute appendicitis, 16 patients (25%) had an appendicolith, 64 patients (100%) had positive fat stranding, 39 patients (60%) had free fluid, 27 patients (42%) had thickened paracolic gutter fascia and 35 patients (54%) had thickened cecal pole/terminal ileum.

**Table 2:** Imaging results of patients for US and CT imaging (N= 351). US: Ultrasound, CT: Computed Tomography.

Imaging Technique	Result	Count	Percentage, %
US imaging	Positive	22	6.3
	Negative	54	15.4
	Inconclusive	152	43.3
	Not performed	123	35.0
CT imaging	Positive	79	22.5
	Negative	141	40.2
	Inconclusive	6	1.7
	Not performed	125	35.6

Post-appendectomy pathology results revealed that 64 out of 351 patients were diagnosed with AA in which 18 of those confirmed patients underwent US imaging (Table 3). Using pathology results as a reference, 12 out of 14 positive patients (85.7%) are true positive while two out of four negative patients (50.0%) are true negative, as confirmed by pathology results. On the other hand, two out of 14 positive patients (14.3%) are false positives while two out of four negative patients (50.0%) are false negatives which implies an incorrect diagnosis.

**Table 3:** Comparison of US imaging results to the pathology results. US: Ultrasound.

Variable		Total	Pathology Results	
			Yes	No
Positive	Count	14	12	2
	%within US result	100.0%	85.7%	14.3%
	%within Pathology result	77.8%	85.7%	50.0%
	% of Total	77.8%	66.7%	11.1%
Negative	Count	4	2	2
	%within US result	100.0%	50.0%	50.0%
	%within Pathology result	22.2%	14.3%	50.0%
	% of Total	22.2%	11.1%	11.1%
Total	Count	18	14	4
	%within US result	100.0%	77.8%	22.2%
	%within Pathology result	100.0%	100.0%	100.0%
	% of Total	100.0%	77.8%	22.2%

Moreover, 62 of those confirmed AA patients underwent CT imaging (Table 4). Forty-six out of 49 positive patients (93.9%) are true positive while nine out of 13 negative patients (69.2%) are true negative, as confirmed by pathology results. On the other hand, only three out of 49 positive patients (6.1%) were false positive while only four out of 13 negative patients (30.8%) were false negative which implies a relatively lower number of incorrect diagnoses.

**Table 4:** Comparison of CT imaging results to the pathology results. CT: Computed Tomography

Variable		Total	Pathology Results	
			Yes	No
Positive	Count	49	46	3
	%within CT result	100.0%	93.9%	6.1%
	%within Pathology result	79.0%	92.0%	25.0%
	% of Total	79.0%	74.2%	4.8%
Negative	Count	13	4	9
	%within CT result	100.0%	30.8%	69.2%
	%within Pathology result	21.0%	8.0%	75.0%
	% of Total	21.0%	6.5%	14.5%
Total	Count	62	50	12
	%within CT result	100.0%	80.6%	19.4%
	%within Pathology result	100.0%	100.0%	100.0%
	% of Total	100.0%	80.6%	19.4%

Using the number of patients with surgical-pathological confirmed acute appendicitis, the diagnostic accuracies of using US and CT imaging were compared to the obtained pathology results (Table 5). For US imaging, at a 95% confidence interval, the sensitivity and specificity were computed at 85.71% and 50.00%, respectively. For CT imaging, at a 95% confidence interval, the sensitivity and specificity were computed at 92.00% and 75.00%, respectively. The PPV and NPV were 93.88% and 69.23%, respectively.

**Table 5:** Statistical analysis of determining acute appendicitis. CI: Confidence Interval at 95%, * Dependant on disease prevalence

Statistic	Values (CI)	
	US imaging	CT imaging
Sensitivity	85.71 (57.19-98.22)	92.00 (80.77-97.78)
Specificity	50.00 (6.76-93.24)	75.00 (42.81- 94.51)
Disease prevalence*	77.78 (52.36-93.59)	80.65 (68.63-89.58)
Positive predictive value*	85.71 (68.76-94.24)	93.88 (85.15- 97.62)
Negative predictive value*	50.00 (16.60-83.40)	69.23 (45.41-85.89)
Accuracy*	77.78 (52.36-93.59)	88.71 (78.11-95.34)
Positive likelihood ratio	1.71 (0.63- 4.67)	3.68 (1.38-9.84)
Negative likelihood ratio	0.29 (0.06-1.44)	0.11 (0.04- 0.29)

## Discussion

Acute appendicitis, produced via appendix inflammation, has been considered as the most common etiological factor for abdominal pain with a lifetime prevalence of 7%. Despite its prevalence, clinical diagnosis of AA remains challenging and needs the importance of imaging modalities in the detection of AA among suspected patients. However, the decision to choose which diagnosing technique should be used remains challenging [4]. US imaging is widely used as the basic diagnostic technique and is usually the continuation of the clinical examinations for suspected AA patients. It is considered a more viable choice for children but usually gave an indistinct result for older patients. Some studies also revealed that there was a significant difference in the US results of obese and non-obese patients due to the interferences caused by an extreme proportion of extra-mesenteric fats [9]. From these, CT imaging is considered as a more applicable choice to some patients like obese and older individuals in which US imaging is not that clear. Its higher sensitivity, which approaches 100%, is clearly an advantage but its costs and the ionizing radiation exposure are some of its drawbacks [4,7]. With an estimated risk of 2%, the exposure to radiation that may induce cancer was addressed by introducing MRI as an alternative technique that can also give high accuracy in determining AA [10-12]. Considering several factors, such as accuracy, availability, and safety, any of these imaging techniques may be used to avoid delay in the diagnosis of AA [13]. Nonetheless, CT has been used as the reference standard, on top of US and MRI, for determining appendicitis since the start of the millennium [7,12].

The diagnostic accuracies of both US and CT imaging in diagnosing AA were compared. The US accuracy was 77.78%, sensitivity was 85.71% and specificity was 50.00%. Similarly, Farooq and co-workers (2020) reported a comparable US accuracy of 77.5%, sensitivity of 80%, and specificity of 60% [14]. Ahmed and co-workers (2016) also demonstrated comparable findings with a US accuracy of 84%, sensitivity of 86%, and specificity of 80% [10]. On the other hand, Alelyani and co-workers (2021) reported some inconsistent trends in which the US accuracy and sensitivity are relatively lower at 46.2% and 38.9%, respectively, but with a specificity of 89.5% [15].

Comparing US to CT imaging, this study showed better performance for CT imaging which was 88.71% accurate, 92.00% sensitive, and 75.00% specific. The PPV and NPV of US imaging were also lower than that of CT imaging. The respective values were 85.71% and 50.00% for US imaging while 93.88% and 69.23% for CT imaging. These findings were in line with the results obtained by Hwang (2018) as the diagnostic pooled values for sensitivity, specificity, PPV, and NPV in US imaging were 86%, 94%, 100%, and 92%, respectively. Meanwhile, the respective values for CT imaging were 95%, 94%, 95%, and 99% [16]. Consequently, Alshebromi and co-workers (2019) reported that US imaging has a sensitivity of 37.0% and specificity of 100.0% while CT imaging has 86.0% and 16.7%, respectively [8]. Reich and co-workers (2011) also compared US and CT imaging in terms of sensitivity and PPV in which results revealed that CT had better sensitivity and PPV of both 100% compared to that of US with sensitivity and PPV of 68.4% and 94.5%, respectively. The negative appendectomy rate (NAR) was also compared, and a significant difference was seen between the 0% NAR of CT imaging and 5.5% of US imaging, reported after positive imaging [17]. Another study that demonstrated the effect of BMI on the accuracy of US and CT in diagnosing appendicitis in children also revealed that CT has better sensitivity of 96% and specificity of 97% compared to that of US with an overall sensitivity of 38% and a comparable specificity of 97%. It was also observed that CT gave excellent accuracy regardless of BMI while US has a decreasing sensitivity with an increasing BMI [6]. The same findings regarding the accuracy of the two were also obtained by van Randen and co-workers (2011) in which the sensitivity of CT was higher at 94% compared to the 76% sensitivity of US imaging [18]. Another prospective study also directly compared the two and the results agreed that CT was superior to US. The obtained CT sensitivity was 100% and specificity was 100%, while the US sensitivity was 91% and specificity was 98% only [19]. A systematic review, conducted by Karul and co-workers (2014), revealed that US imaging also had lower sensitivity and specificity than CT imaging, in general. For US imaging, the pooled sensitivity and specificity values ranged from 71.2-92.0% and 83.3-96.6%, respectively. For CT imaging, the respective pooled values ranged from 89.0-100.0% and 89.0-98.0% [7].

Despite the alignment with the related literature, US imaging has still been extensively used even if its accuracy in diagnosing AA was not high compared to CT imaging. Its non-invasiveness and cost-effectiveness have been its principal advantage against the latter. However, the consistent quality examination and reproducibility of CT scanning can provide a more accurate diagnosis, which may lower diagnosis confusion, delay treatment, and even mortality. With these, it is recommended that inconclusive US imaging results should be verified via CT imaging [5,7].

Limitations

This study has potential limitations to be considered. First, the body-mass-index (BMI) of the patients was not studied. It is well known that US has the difficulty in penetrating the fat; thus, the US imaging results might be equivocal for obese patients, in case there is any. Second, the sensitivity of US imaging might vary and might be underestimated depending on the sonographer who administered the results. Being used for initial and urgent diagnosis, US is usually performed and interpreted by an unsupervised radiological resident rather than an attending radiologist. In some literature, it was found that US results performed by an unsupervised resident gave a significantly lower sensitivity than that interpreted by an attending radiologist [20]. Lastly, the age bracket of <20 years old was a wide range and may partially contribute to an adult population; thus, the study cannot be generalized to children.

## Conclusions

This study demonstrated a comparison of the effectiveness of US and CT imaging in determining acute appendicitis with reference to pathology results. CT imaging was found to be more effective and accurate than US imaging. Although US imaging is cost-effective and non-invasive, the high diagnostic accuracy of CT imaging made the latter a more effective tool in appendicitis management resulting to prompt treatment identification, unnecessary appendectomies, and fewer hospital admissions.

## References

[1] Moris D, Paulson EK, Pappas TN (2021). Diagnosis and management of acute appendicitis in adults: a review. JAMA.

[2] Petroianu A (2012). Diagnosis of acute appendicitis. Int J Surg.

[3] Di Saverio S, Podda M, De Simone B (2020). Diagnosis and treatment of acute appendicitis: 2020 update of the WSES Jerusalem guidelines. World J Emerg Surg.

[4] Naidu A, Dhruwa C (2022). Study on evaluation of ultrasound and CT scan in diagnosis of appendicitis. Int J Health Sci.

[5] Sheeba A, Sahito AA, Jatoi A, Naz F, Gul N, Yousif S (2021). Diagnostic accuracy of ultrasound in the detection of acute appendicitis by taking CT abdomen as gold standard. Pakistan Journal of Medical & Health Sciences.

[6] Abo A, Shannon M, Taylor G, Bachur R (2011). The influence of body mass index on the accuracy of ultrasound and computed tomography in diagnosing appendicitis in children. Pediatr Emerg Care.

[7] Karul M, Berliner C, Keller S, Tsui TY, Yamamura J (2014). Imaging of appendicitis in adults. Rofo.

[8] Alshebromi MH, Alsaigh SH, Aldhubayb MA (2019). Sensitivity and specificity of computed tomography and ultrasound for the prediction of acute appendicitis at King Fahad Specialist Hospital in Buraidah, Saudi Arabia. Saudi Med J.

[9] Morrow SE, Newman KD (2007). Current management of appendicitis. Semin Pediatr Surg.

[10] Haider AH, Qureshi MN, Bila R, Ahmed AH (2016). Acute appendicitis; ultrasonography as pre-operative screening tool. Pak Armed Forces Med J.

[11] Berrington de González A, Mahesh M, Kim KP, Bhargavan M, Lewis R, Mettler F, Land C (2009). Projected cancer risks from computed tomographic scans performed in the United States in 2007. Arch Intern Med.

[12] Kim DW, Suh CH, Yoon HM, Kim JR, Jung AY, Lee JS, Cho YA (2018). Visibility of normal appendix on CT, MRI, and sonography: a systematic review and meta-analysis. AJR Am J Roentgenol.

[13] Bhangu A, Søreide K, Di Saverio S, Assarsson JH, Drake FT (2015). Acute appendicitis: modern understanding of pathogenesis, diagnosis, and management. Lancet.

[14] Farooq A, Zameer S, Khadim R (2020). Diagnostic accuracy of ultrasound in acute appendicitis in comparison with alvarado score keeping histopathological correlation as gold standard. Pak Armed Forces Med J.

[15] Alelyani M, Hadadi I, Shubayr N (2021). Evaluation of ultrasound accuracy in acute appendicitis diagnosis. Appl Sci.

[16] Hwang ME (2018). Sonography and computed tomography in diagnosing acute appendicitis. Radiol Technol.

[17] Reich B, Zalut T, Weiner SG (2011). An international evaluation of ultrasound vs. computed tomography in the diagnosis of appendicitis. Int J Emerg Med.

[18] van Randen A, Laméris W, van Es HW (2011). A comparison of the accuracy of ultrasound and computed tomography in common diagnoses causing acute abdominal pain. Eur Radiol.

[19] Toorenvliet BR, Wiersma F, Bakker RF, Merkus JW, Breslau PJ, Hamming JF (2010). Routine ultrasound and limited computed tomography for the diagnosis of acute appendicitis. World J Surg.

[20] Laméris W, van Randen A, van Es HW (2009). Imaging strategies for detection of urgent conditions in patients with acute abdominal pain: diagnostic accuracy study. BMJ.

